# Clinical Remarks on Coup De Soleil

**Published:** 1860-10

**Authors:** J. F. Meigs

**Affiliations:** Attending Physician


*Clinical Remarks on Coup de Soleil.* By J. F. Meigs, M. D., Attending Physician.

This affection is variously known by the names of *Ictus Solis* sun stroke, heat asphyxia. *Symptoms.*—It is characterized by a hot dry skin; a sense of constriction of the chest with labored breathing, great prostration, with inability to answer questions without weeping, even in the strongest and most robust; tumultuous action of the heart, with strong pulsation of the carotids; pulse very variable, never full and hard; headache, especially on the top of the head; the conjunctiva injected ; pupils acting to the light, unless during convulsions or coma, when they are fixed and contracted (in several instances they have been observed to dilate suddenly, after having been fixed and contracted); the countenance is generally pale at the commencement, but in several instances (fatal cases) it assumed a leaden hue the urine is never entirely suppressed, but it passed off involuntarily, guttatim; the bowels are generally costive, but occasionally are perfectly natural. There is also a great desire to sleep; so much so, that if not checked, it passes into coma, which almost invariably terminates in death. During the coma, loud moaning is always present. Death ensues instantly, or from coma. Sometimes the patient is seized with maniacal symptoms; at others, he laughs unnaturally, then becomes alarmed and excited if spoken to, and any attempt at deglutition brings on convulsions.

*Post-mortem Examination.*—The chief abnormal appearance is an excess of venous blood in the brain and congestion of the lungs and liver. Dr. Boisliniere, Coroner of St. Louis, in a report of seventy-two cases examined by him and published in the Medical and Surgical Journal of that city, says, the necropsies revealed the following conditions:

*External Appearances.*—Marked lividity of the skin; neck and anterior part of the chest became soon of a purple or blue color; in a few hours the abdomen was quite tympanitic, an abundant froth came out of the mouth and nostrils, resembling thick lather, mixed sometimes with a little blood. By pressing upon the chest, blood could be made to flow freely from the mouth and nostrils. The lungs and heart were in every case seen to be more or less congested; the right side of the heart and the pulmonary artery generally contained black and liquid blood; left side empty; on section, the lungs were found to contain an abundant quantity of frothy mucus, mixed with more or less arterialized blood. By moderate pressure on the chest, as above observed, this bloody froth could be made to run out freely from the mouth and nostrils. So characteristic was this appearance, that from its presence alone, many post-mortem examinations towards the end of the summer were dispensed with, the jury, after a short explanation, being able to make a satisfactory verdict of death by sun-stroke. The brain was generally found normal; in a few cases only, there was moderate congestion of the superior cerebral veins and of the sinuses.—This, the author accounts for, by the difficulty the blood found in returning from the thoracic organs, already full of venous blood. The liver and spleen were, as a rule, enlarged; the latter particularly, softened. Dr. B. regards as the cause of this affection, a hot and rarified atmosphere, want of oxygen; as he says, the disease occurs in the house as well as when exposed to the sun. Hence he concluded that rarified, or poorly oxygenated air, is the "*conditio sine qua non*" of sun-stroke."

*Treatment.*—The chief point is to arouse the nevous system, which is best accomplished by pouring cold water from a height over the head and the nape of the neck, and dashing it over the face and chest as long as there is any tendency to coma or sleep. Sometimes, the patient may be roused by speaking to him and shaking him, if necessary. When he can swallow, brandy and ammonia may be administered liberally. The Indian surgeons give croton oil and calomel to act on the liver and move the bowels. At this institution, the turpentine injection is given. For the weight and distress at the scrobiculus cordis, the best means will be the rubbing of turpentine for some time over the chest and stomach, with sinapisms to the legs and stimulating enemata. The after treatment consists in the employment of good nourishment and stimulants, as arrowroot, beef tea, wine, brandy, and ammonia; cold to the head, blisters to the nucha, and acting on the liver and the bowels. The patient is not to be considered out of danger until his skin has become cool and moist. Venesection has been found almost, if not always, injurious and fatal by the Indian surgeons. Cupping to the back of the neck, when external nervous congestion is marked, has sometimes been thought useful.—But two cases had occurred this summer at the hospital, one of which was fatal in fifteen minutes, and presented all the appearances as mentioned by Dr. B. The other case was a young man, aged 20, very healthy, brought in August 11th, and had perfectly recovered by the 13th. The day previous, he had had a slight diarrhœ. While engaged at work on the roof of a house, his eyes became dim, he felt very weak, and fell into a state of insensibility. He was given immediately upon his admission ℞ Spt. Vini Gall., Aquæ *aa* f ℥ ss., Ammonii Aromat ʒ j. M. Half of this, every fifteen minutes. Along with this, an enema of turpentine f ℥ ss. Saponis ℥ j., Aquæ Oj. was given, and sinapisms were ap- to the epigastrium. He rallied very speedily, and was discharged cured on the 15th.—*American Medical Times.*

